# Endoplasmic Reticulum Isolation: An Optimized Approach into Cells and Mouse Liver Fractionation

**DOI:** 10.21769/BioProtoc.4803

**Published:** 2023-09-05

**Authors:** Marc Leiro, Raúl Ventura, Nil Rojo-Querol, María Isabel Hernández-Alvarez

**Affiliations:** 1 Departament de Bioquímica i Biomedicina Molecular, Facultat de Biologia, Universitat de Barcelona, Barcelona, Spain; 2 Institut de Biomedicina de la Universitat de Barcelona (IBUB), Barcelona, Spain; 3 Centro de Investigación Biomédica en Red de Diabetes y Enfermedades Metabólicas Asociadas (CIBERDEM), Instituto de Salud Carlos III, Madrid, Spain

**Keywords:** Endoplasmic reticulum (ER), Organelle isolation, Cells, Mouse liver, Subcellular fractionation, Mitochondrial-associated membranes (MAMs), Time efficient

## Abstract

The subfractionation of the endoplasmic reticulum (ER) is a widely used technique in cell biology. However, current protocols present limitations such as low yield, the use of large number of dishes, and contamination with other organelles. Here, we describe an improved method for ER subfractionation that solves other reported methods' main limitations of being time consuming and requiring less starting material. Our protocol involves a combination of different centrifugations and special buffer incubations as well as a fine-tuned method for homogenization followed by western blotting to confirm the purity of the fractions. This protocol contains a method to extract clean ER samples from cells using only five (150 mm) dishes instead of over 50 plates needed in other protocols. In addition, in this article we not only propose a new cell fractionation approach but also an optimized method to isolate pure ER fractions from one mouse liver instead of three, which are commonly used in other protocols. The protocols described here are optimized for time efficiency and designed for seamless execution in any laboratory, eliminating the need for special/patented reagents.

Key features

• Subcellular fractionation from cells and mouse liver.

• Uses only five dishes (150 mm) or one mouse liver to extract highly enriched endoplasmic reticulum without mitochondrial-associated membrane contamination.

• These protocols require the use of ultracentrifuges, dounce homogenizers, and/or Teflon Potter Elvehjem.

As a result, highly enriched/clean samples are obtained.

Graphical overview

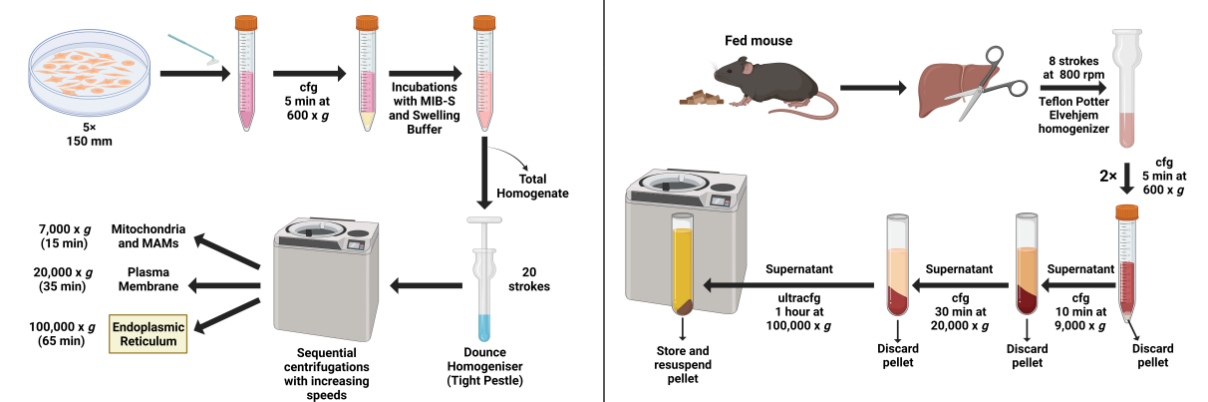

## Background

The endoplasmic reticulum (ER) is a continuous membrane system that forms a series of flattened sacs within the cytoplasm of eukaryotic cells and serves multiple functions. Some of its most relevant roles include the synthesis, folding, modification, and transport of proteins (Schwarz and Blower, 2016). The ER was first observed by electron microscopy in 1945 by Porter and Claude (Porter et al., 1945), but its isolation as a distinct organelle was not achieved until 1959 by Palade and Siekevitz (Siekevitz and Palade, 1959). In this study, authors used differential centrifugation to separate ER membranes from other cellular components. This method was later refined by adding density gradient centrifugations to obtain cleaner (non-other contaminating organelles marker) fractions (Lee et al., 2010).

The isolation of the ER has facilitated and enhanced the understanding of its structure, function, and interactions with other organelles. Some of these studies revealed that the ER forms contact sites with other membranes, such as plasma membrane–associated membranes (PAMs) (Suski et al., 2014) and mitochondria-associated membranes (MAMs) (Missiroli et al., 2018), which mediate transport of proteins, lipids, and metabolites (Ventura et al., 2022).

However, despite being a highly common technique, the isolation of ER has some limitations. Firstly, it requires a large amount of starting material (usually several grams of tissue or cell number) and takes a long time to complete (6 h in cells because of the time needed to recover the starting material from the plates). Secondly, existing protocols may not be suitable for all tissues or cell types that have different membrane properties or distributions. Thirdly, it is quite difficult to obtain pure samples, and thus achieving highly enriched fractions can be challenging.

Most already existing methods are able to isolate ER indirectly, but only a very small number of protocols are dedicated to the extraction of this particular organelle (Croze and Morré, 1984). This scenario can be found in some of the published protocols that use mouse liver, such as the ones described by Wieckowski et al. (2009) or Suski et al. (2014), where they propose a method to isolate specific areas of the ER, such as MAMs and PAMs, respectively. This fact can also be a limiting factor.

In regard to HeLa cells, no protocols were found to specifically extract the ER as well. Moreover, some protocols that work with similar adherent cells require a large number of dishes (over 50 dishes) (Wieckowski et al., 2009; Williamson et al., 2015), which complicates the isolation and the previous cell culture work. Additionally, a similar situation happens with subcellular fractionations that use liver tissue. According to published protocols, fractionations of liver tissue are performed using rat liver rather than mouse liver, and they usually require from 8 to 10 g of tissue (Suski et al., 2014). When extrapolated to a mouse model, five or six animals for every subfractionation are needed.

Since our laboratory is mainly focused on the study of mitochondrial dynamics, ER–mitochondria contacts, and its relationship with metabolic diseases (Hernández-Alvarez et al., 2019), a protocol that overcame some of these limitations was necessary. Hence, in this article, we provide an optimized and upgraded version of already existing protocols for ER isolation achieving a high throughput using the minimum amount of starting sample. Furthermore, the protocols described below are optimized for time efficiency and designed for seamless execution in any laboratory, eliminating the need for special/patented reagents. Other protocols do not report the yield of ER.

## Materials and reagents

**Biological materials**

1. Mouse liver or cell line of interest

**Antibodies**

1. Mouse monoclonal anti-PDI (C-2) (Santa Cruz Biotechnology, catalog number: sc-74551)
2. Rabbit monoclonal anti-Tom20 (D8T4N) (Cell Signalling Technology, catalog number: 42406)
3. Mouse monoclonal anti-VDAC1 (B-6) (Santa Cruz Biotechnology, catalog number: 390996)
4. Mouse monoclonal anti-Tim23 (H-8) (Santa Cruz Biotechnology, catalog number: sc-514463)
5. Rabbit monoclonal anti-FACL4 (EPR8640) (Abcam, catalog number: ab155282)
6. Mouse monoclonal anti-Na^+^/K^+^ ATPase alpha 3 subunit (Sigma-Aldrich, catalog number: 05-369-25UG)

**Reagents**

**Isolation from cells**

1. HEPES (Merck, Sigma-Aldrich, catalog number: 54461)
2. D-Sucrose (Thermo Fisher Scientific, Fisher bioreagents, catalog number: BP220-1)
3. EGTA (Merck, Calbiochem, catalog number: 324626)
4. KCl (Honeywell, Fluka analytical, catalog number: 31248)

**Isolation from mouse liver**

1. D-Mannitol (Merck, Calbiochem, catalog number: 443907)
2. D-Sucrose (Thermo Fisher Scientific, Fisher bioreagents, catalog number: BP220-1)
3. Albumin from bovine serum (BSA) (Merck, Sigma-Aldrich, catalog number: A7906)
4. EGTA (Merck, Calbiochem, catalog number: 324626)
5. Tris hydrochloride (Tris-HCl) for buffer solutions (PanReac AppliChem, catalog number: A1087)

**10× PBS**

1. NaCl (, catalog number)
2. Na_2_HPO_4_·2H_2_O (Thermo Fisher Scientific, catalog number: 15613040)
3. KH_2_PO_4_ (Merck, catalog number: 104873)
4. KCl (Honeywell, Fluka analytical, catalog number: 31248)

**Solutions**

1. Microsome isolation buffer cells (MIB-C) (see Recipes)
2. Microsome isolation stability buffer (MIB-S) (see Recipes)
3. 5× swelling buffer (SB) (see Recipes)
4. Microsome isolation buffer liver (MIB-L) (see Recipes)
5. 10× PBS (see Recipes)

**Recipes**

1. **Microsome isolation buffer liver (MIB-L)**
  **Table:** 

Reagent	Final concentration	Quantity
D-Mannitol	225 mM	0.41 g
D-Sucrose	75 mM	0.26 g
EGTA (100 mM; pH 8)	0.5 mM	0.05 mL
Tris-HCl (1 M, pH 6.5)	30 mM	0.3 mL
BSA	0.5%	0.05 g
MQH_2_O	n/a	Up to 10 mL
Total	n/a	10 mL
2. **Microsome isolation buffer cells (MIB-C)**
  **Table:** 

Reagent	Final concentration	Quantity
Tris-HCl (1 M, pH 6.5)	30 mM	450 μL
D-Mannitol	225 mM	0.61 g
D-Sucrose	75 mM	0.38 g
MQH_2_O	n/a	Up to 15 mL
Total	n/a	15 mL
3. **Microsome isolation stability buffer (MIB-S)**
  **Table:** 

Reagent	Final concentration	Quantity
D-Sucrose	0.25 M	1.2 g
KCl	25 mM	0.028 g
HEPES (1 M)	10 mM	150 μL
EGTA (100 mM; pH 8)	1 mM	150 μL
MQH_2_O	n/a	Up to 15 mL
Total	n/a	15 mL
4. **5× swelling buffer (SB)**
  **Table:** 

Reagent	Final concentration	Quantity
KCl	125 mM	0.14 g
HEPES (1 M)	50 mM	750 μL
EGTA (100 mM; pH 8)	5 mM	150 μL
MQH_2_O	n/a	Up to 15 mL
Total	n/a	15 mL
5. **10× PBS**
  **Table:** 

Reagent	Final concentration	Quantity
NaCl	1.5 M	400 g
Na_2_HPO_4_·2H_2_O	0.1 M	80 g
KH_2_PO_4_	15 mM	10 g
KCl	25 mM	10 g
MQH_2_O	n/a	up to 5 L
Total	n/a	5 L

## Equipment

1. Corning^®^ tissue culture–treated culture dishes (150 mm × 25 mm) (Merck, Corning^®^, catalog number: CLS430599)
2. Active Motif dounce homogenizer (Fisher Scientific, Active Motif 40401, catalog number: NC0569256)
3. Nunc^TM^ cell scrapers (Thermo Scientific^TM^, catalog number: 179707PK)
4. L-90K ultracentrifuge (Beckman coulter, catalog number: 8043-30-1191)
5. Type 90 Ti fixed-angle titanium rotor (Beckman coulter, catalog number: 355530)
6. Centrifuge 5810R (Eppendorf, catalog number: 5811000015)
7. 15 mL polypropylene centrifugation tube (Thermo Scientific^TM^, catalog number: 17627105)
8. 8.9 mL ultracentrifuge tube (Beckman coulter, catalog number: 361660)
9. Heidolph RZR 2051 control homogenizer (Fisher Scientific, catalog number: FIS13-880-115)
10. Tissue grinders, Potter Elvehjem type with PTFE pestle for soft tissue, 15 cm^3^ (Avantor, VWR^®^, catalog number 432-5041)
11. Western blotting equipment (Bio-Rad)

## Procedure

- **ER isolation from HeLa cells (Figure 1)**
  **Figure 1.**
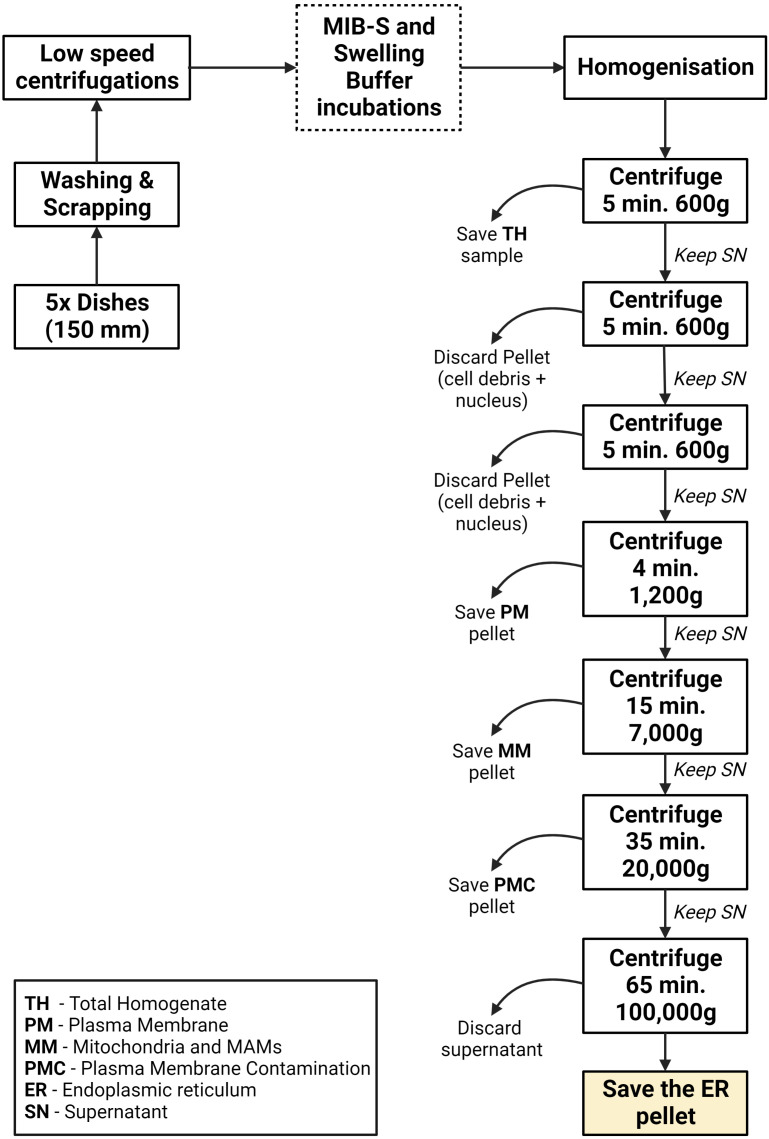
Graphical summary of the endoplasmic reticulum (ER) isolation process using HeLa cells
  1. Cell collection, rinsing, and scraping
    - For this protocol, five 150 mm dishes are scraped one at a time directly on the laboratory bench on ice or a cold surface (at 4 °C). In the meantime, while one dish is scrapped, the other dishes must remain in the incubator at 37 °C. Before scraping, aspirate the media and then add an approximate volume of 8 mL of PBS 1× to rinse the dish. After gently swirling to ensure the PBS covers the entire surface of the dish, aspirate the PBS and then add 4.5 mL of ice-cold MIB-S buffer. Once the MIB-S buffer is added, the dish is ready to get scrapped with a manual scraper, just in one direction.
      ** Important step:** The MIB-S buffer must be kept cold to ensure its proper functioning and facilitate the process.
*Notes:*
*i. Ensure that all surfaces and equipment are disinfected with 70% ethanol before use.*
*ii. A standard cell culture incubator with controlled CO_2_ levels should be used. If not possible, use a 37 °C incubator (without CO_2_ control), ensuring that cells are kept only for a short period of time to prevent their unwanted detachment.*
    - As a result of the first scraping, a dish containing a cell suspension in 4.5 mL of buffer will be obtained. At this point, the next dish will be rinsed as explained in previous steps and, instead of adding an additional 4.5 mL of buffer, the cell suspension of the first dish will be reused to scrape the following dish.
      ** Important step:** The 4.5 mL of MIB-S buffer is reused along all the five dishes. Hence, the same volume of buffer will be used to accumulate the cells from all the dishes.
*Note: For this step, trypsin can also be used to detach and collect the cells. In this case, cells must be centrifuged, rinsed with PBS, and later resuspended with 4.5 mL of MIB-S buffer.*
    - Finally, transfer the cell suspension to a 15 mL polypropylene centrifugation tube.
*Note: The final volume obtained is expected to be approximately 6–8 mL due to the accumulation of the cells of each five plates.*
  2. Low speed centrifugations and incubations
    - Centrifuge the polypropylene tube containing the cell suspension at 600× *g* for 5 min at 4 °C. The pellet obtained will correspond to the cells collected in the last step.
*Note: This centrifugation and all the following ones are performed at 4 °C as stated in section General notes and troubleshooting.*
    - Measure the approximate volume (usually 0.5 mL) of the cell pellet obtained and prepare three times this volume of SB.
    - Aspirate the supernatant and resuspend the pellet in the volume calculated in the previous step of cold SB. In the case of having 0.5 mL, 1.5 mL of SB will be prepared.
*Note: To resuspend, use a 1 mL Pasteur pipette to prevent breaking the cells.*
    - Incubate the cell suspension at 4 °C for 35 min.
    - Once the incubation is complete, centrifuge again at 600× *g* for 5 min. This time, the resulting pellet will be comprised of swollen cells.
    - Carefully aspirate the supernatant and resuspend with 8 mL of MIB-S buffer to avoid osmotic disequilibrium.
  3. Homogenization
    - To obtain the different fractions, cells must be first homogenized to preserve organelle structure. For this process, pour the 8 mL of cell suspension obtained in step A2f into a dounce homogenizer and homogenize using a tight pestle (using a 15 cm^3^ homogenizer) for 20 strokes.
      ** CRITICAL:** This is an important step, and the success of the protocol hinges on the precision with which it is carried out. For it to work correctly, the pestle must be pushed up and down while twisted (or rotated) simultaneously in the same motion. It is extremely important to perform this movement carefully in order to avoid the formation of bubbles.
*Note: Before using the homogenizer and its pestle, rinse them with normal and ultra-pure water. Homogenization must be done on ice to minimize protease effects.*
    - The result from the homogenization will be called *Total Homogenate.* Store a sample of approximately 150 μL at -20 °C to use as a control for the western blot analysis.
  4. Low speed centrifugations: cell debris removal
    - Transfer the Total Homogenate to a 15 mL polypropylene centrifugation tube and centrifuge at 600× *g* for 5 min. The pellet obtained after centrifugation will correspond to cell debris and will be discarded.
    - Transfer the supernatant obtained in the last step to a new 15 mL polypropylene centrifugation tube and centrifuge again at 600× *g* for 5 min. This step is used to further clean the sample. As in the previous step, only the supernatant is kept.
  5. Obtaining the main fractions
    - Transfer the supernatant obtained in step A4b to a new 15 mL polypropylene centrifugation tube and centrifuge at 1,200× *g* for 4 min. After this centrifugation, the resulting pellet will correspond to a sample highly enriched in the plasma membrane. In order to keep this fraction, transfer the supernatant to a new 8.9 mL ultracentrifuge tube and resuspend the pellet in 150 μL of MIB-C buffer. Store this sample at -20 °C for further western blot analysis and label it as *Plasma Membrane* sample.
*Note: After this step, every centrifugation is performed in an ultracentrifuge using the 90Ti fixed-angle rotor from Beckmann.*
    - Centrifuge the supernatant at 7,000× *g* for 15 min. The result from this centrifugation will be a pellet that corresponds to mitochondria and mitochondrial-associated membranes (MAMs) enriched fraction. As in the previous step, transfer the supernatant to a new 8.9 mL ultracentrifuge tube and resuspend the pellet in 150 μL of MIB-C buffer. Store the resuspended pellet at -20 °C for further western blot analysis and label it as *Mitochondria and MAMs* sample.
*Note: It is possible to further isolate pure mitochondria and pure MAMs from this fraction by following the protocol described by Wieckowski et al. (2009).*
    - Centrifuge the supernatant from the previous step at 20,000× *g* for 35 min. The result from this centrifugation will be a pellet that is mainly composed of plasma membrane contamination. As previously mentioned, just like in the previous step, transfer the supernatant to a new 8.9 mL ultracentrifuge tube and resuspend the pellet in 150 μL of MIB-C buffer. Store the resuspended pellet at -20 °C for further western blot analysis and label it as *Plasma Membrane Contamination* sample.
    - Centrifuge the supernatant from the previous step at 100,000× *g* for 65 min. Finally, the result from this centrifugation will be a highly enriched endoplasmic reticulum pellet. To store the sample, aspirate the supernatant and resuspend the endoplasmic reticulum pellet in 150 μL of MIB-C at -20 °C. *Results of this fractionation as well as a graphical guide of this protocol can be observed in Figure 2 and Figure 3.*
      **Figure 2.**
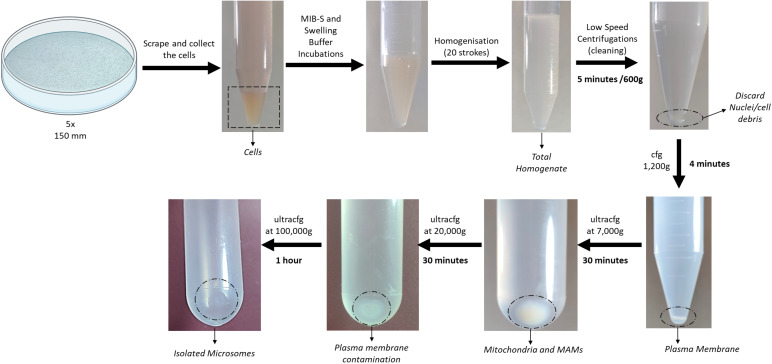
Graphical overview of key steps in the subcellular fractionation protocol using HeLa cells
      **Figure 3.**
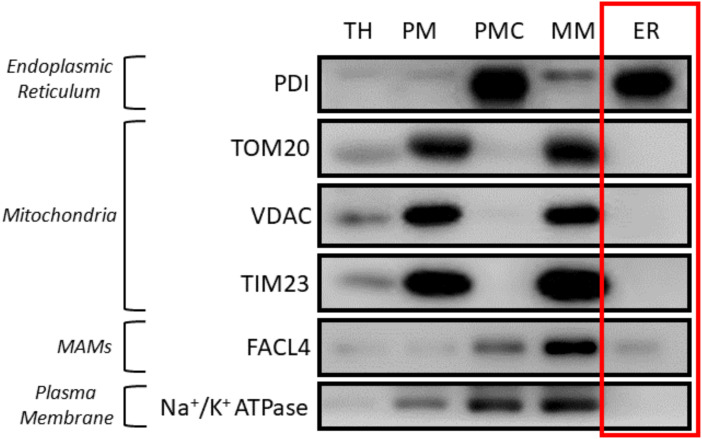
Western blot results of subcellular fractionation protocol using HeLa cells. For this validation, 20 μg of protein were loaded in a 10% SDS-PAGE (sodium dodecyl sulphate polyacrylamide gel electrophoresis) gel. Each fraction was tested with antibodies against specific proteins using a 1:1,000 dilution. Secondary antibodies were also diluted following the same ratio in 5% milk diluted in PBS. In relation to the proteins tested, endoplasmic reticulum (PDI), mitochondria (outer membrane: TOM20, VDAC/inner membrane: Tim23), mitochondria-associated membranes (MAMs) (FACL4) and plasma membrane (Na^+^/K^+^ ATPase) were tested. These proteins were specifically chosen as they are markers of typically contaminating organelles in these protocols. TH: total homogenate; PM: plasma membrane; PMC: plasma membrane contamination; MM: mitochondria and MAMs; ER: endoplasmic reticulum.
- **ER isolation from HeLa cells: adding more dishes**
  When conducting experiments that require a larger pure ER sample size, it may be necessary to add more dishes. However, it is important to keep in mind that as more dishes were added, more contamination (meaning the presence of less pure fractions, not in terms of microorganisms) was observed along our experiments. According to our analyses, the cleanest sample is obtained using five dishes, with the possibility of increasing this to 10, with the following considerations:
  1. 4.5 mL of MIB-S Buffer 1× was used per each set of five dishes. This means that, in this case, a total of 9 mL of this buffer were used only in the scraping part.
  2. Instead of using 8 mL of MIB-S buffer 1× for resuspension in step A3a, 16 mL of buffer was used instead.
  3. Bigger polypropylene centrifugation tubes were used.
  4. In section 5, the total volume was split into two ultracentrifuge tubes and centrifuged using the same rotor as described in the last section. Each pellet was resuspended using 100 μL of MIB-C and both resuspensions were added together in the end (we will call this *making a pool*).
    Even though we observed that the protocol is still quite stable when using 10 dishes, we believe that this should be further improved. In these kinds of scenarios, we recommend performing several fractionations from five dishes simultaneously and making pools of reticulum. On average, we got to obtain approximately 250–300 μg of endoplasmic reticulum for each five dishes; so, if the quantity needed is 1 mg, a more effective and cleaner approach would be to have 20 dishes and perform this protocol four times simultaneously.
- **ER isolation using different cell lines**
  While the fractionation protocol was originally optimized for HeLa cells, we have observed that it can be adapted to other cell types. The main modification involves adjusting the homogenization parameters, including the type of homogenizer and the number of strokes, to account for differences in membrane hardness. As an example, we successfully isolated reticulum from mouse embryonic fibroblast (MEF) cells using an automatic homogenizer at 4,000 rpm for 20 strokes (Figure 4). These findings demonstrate the flexibility of the protocol and its potential to be tailored to other cell types.
  **Figure 4.**
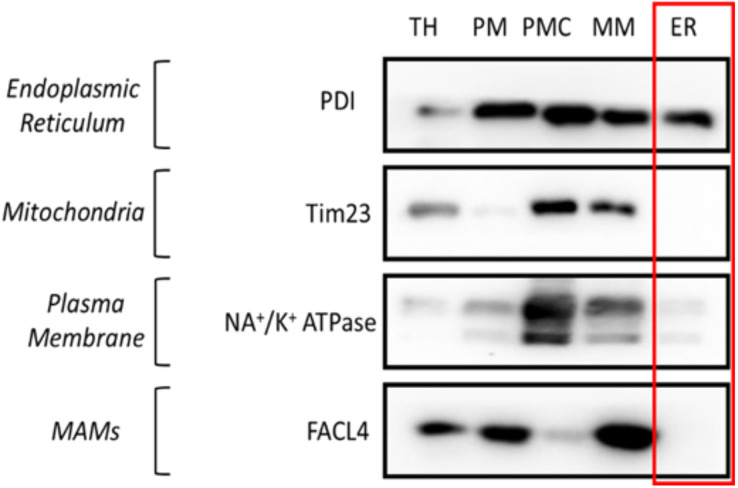
Western blot results of subcellular fractionation protocol using mouse embryonic fibroblast (MEF) cells. For this validation, 20 μg of protein were loaded in a 10% SDS-PAGE (sodium dodecyl sulphate polyacrylamide gel electrophoresis) gel. Each fraction was tested with antibodies against specific proteins using a 1:1,000 dilution. Secondary antibodies were also diluted following the same ratio in 5% milk diluted in PBS. In relation to the proteins tested, endoplasmic reticulum (PDI), mitochondria (Tim23), mitochondria-associated membranes (MAMs) (FACL4), and plasma membrane (Na^+^/K^+^ ATPase) were tested. These proteins were chosen as they are markers of typically contaminating organelles in these protocols. TH: total homogenate; PM: plasma membrane; PMC: plasma membrane contamination; MM: mitochondria and MAMs; ER: endoplasmic reticulum.
- **ER isolation from mouse liver (Figure 5)**
  **Figure 5.**
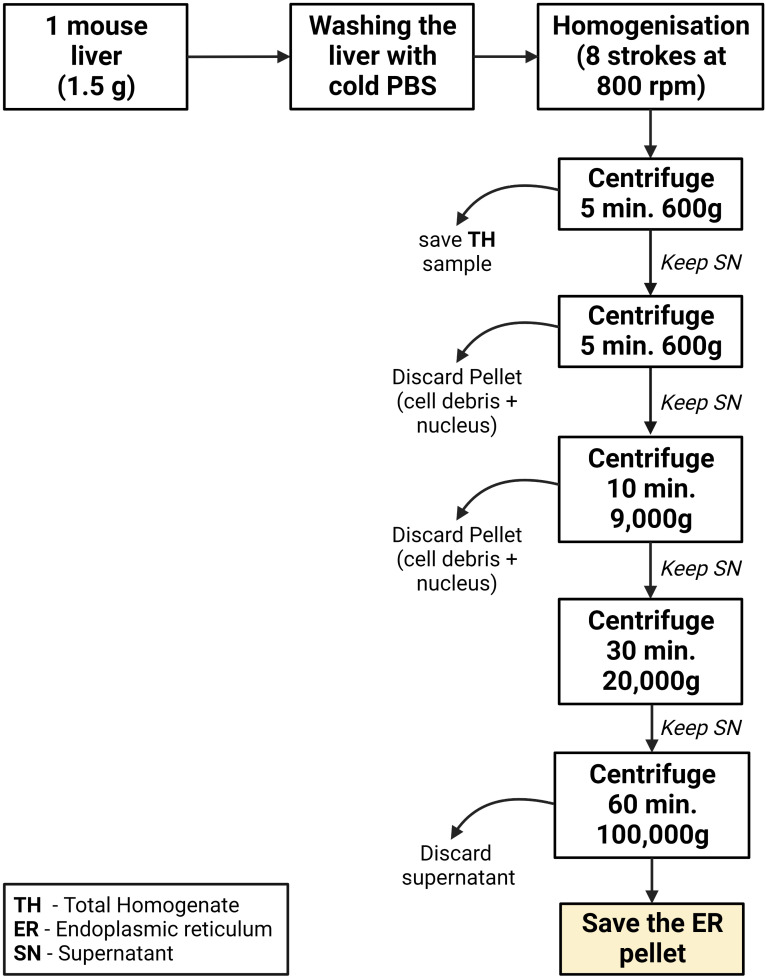
Graphical summary of the ER isolation process using mouse liver
  1. Liver homogenization
    - Euthanize the animal by cervical dislocation, extract the liver in one piece, and remove the gallbladder. Immediately wash the liver two times in ice-cold PBS to remove the blood.
    - Transfer the liver and wash it again with ice-cold PBS. Then, cut the liver into small pieces (0.2 cm approximately) using scissors.
    - Transfer the liver pieces to the 15 mL glass/Teflon Potter Elvehjem homogenizer. Add 4 mL of MIB-L per gram of liver.
*Note: Usually, a normal liver weighs 1.5 g and therefore 6 mL of MIB-L is typically needed. We used the protocol in fed and fast animals, and it worked in both conditions. This was performed in mice fed with a normal diet. The results of this protocol can vary when mice are treated with different diets, especially those that cause alterations in the lipidic composition of the liver. Usually, 500 μL at 15 μg/μL of ER is obtained.*
  2. Low-speed centrifugations
    - Transfer the homogenate to a 15 mL polypropylene centrifugation tube and centrifuge at 600× *g* for 5 min at 4 °C. In this step, nuclei, unbroken cells, and cell debris sediment at the bottom of the tube.
*Notes:*
*i. This centrifugation and all the following ones are performed at 4 °C as stated in General Notes and Troubleshooting.*
*ii. In order to perform a correct ER isolation, it is important to aspirate the lipid before transferring it to a new centrifuge tube. This can be observed as a thin white layer floating on top of the supernatant.*
    - Discard the generated pellet and transfer the supernatant to a new 15 mL polypropylene centrifugation tube. This tube is then centrifuged at 600× *g* for 5 min to remove remaining cell debris.
*Note: As in the previous step, the lipid layer found floating at the top of the tube must be discarded.*
    - Discard the pellet, transfer the supernatant to a new 8.9 mL polypropylene centrifugation tube, and centrifuge it at 9,000× *g* for 10 min using the Beckmann 90-Ti fixed angle rotor. This separates ER (supernatant) from crude mitochondria and MAMs, which in the end are going to be observed as a pellet. This fraction can be saved for analysis purposes as *Mitochondria and MAMs*.
*Note: It is possible to further isolate pure mitochondria and pure MAMs from this fraction by using the protocol described by Wieckowski et al. (2009).*
  3. High-speed centrifugations
    - Centrifuge the supernatant at 20,000× *g* for 30 min using the same rotor as in the previous step. In this case, the pellet will contain mainly plasma membrane and other membranes with similar density characteristics.
*Note: If a lipid layer is formed, discard it as in previous steps.*
    - Transfer the supernatant to a new 8.9 mL polypropylene centrifugation tube and centrifuge at 100,000× *g* for 1 h with the same rotor.
    - Finally, aspirate the supernatant and resuspend the pellet with 500 μL of MIB-L. This pellet corresponds to a highly enriched ER fraction and will be stored at -20 °C for further analysis. Results of this fractionation as well as a graphical guide of this protocol can be observed in Figure 6 and Figure 7.
  **Figure 6.**
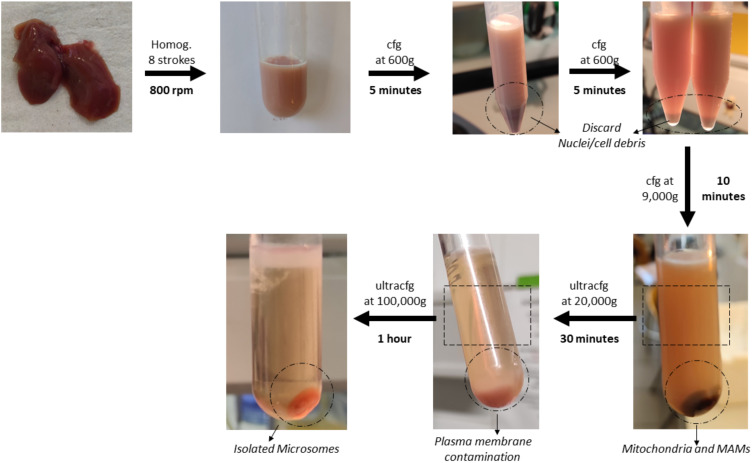
Graphical overview of key steps in the subcellular fractionation protocol using mouse liver
  **Figure 7.**
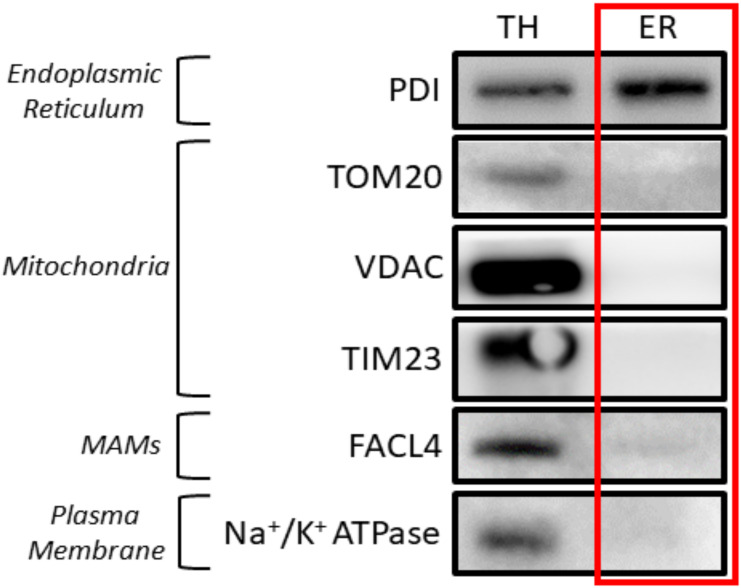
Western blot results of the subcellular fractionation protocol using mouse liver. 20 μg of protein was loaded in a 10% SDS-PAGE (sodium dodecyl sulphate polyacrylamide gel electrophoresis) gel. Each fraction was tested with antibodies against specific proteins using a 1:1,000 dilution. Secondary antibodies were also diluted following the same ratio in 5% milk diluted in PBS. In relation to the proteins tested, endoplasmic reticulum (PDI), mitochondria (outer membrane: TOM20, VDAC / inner membrane: Tim23), mitochondria-associated membranes (MAMs) (FACL4), and plasma membrane (Na^+^/K^+^ ATPase) were tested. These proteins were specifically chosen as they are markers of typically contaminating organelles in these protocols. TH: total homogenate; ER: endoplasmic reticulum.

## Data analysis

In accordance with existing literature, the verification of this technique is qualitative rather than quantitative. Hence, the validity of this technique depends on observing markers specific to ER in the corresponding lanes and ensuring that no other organelles’ markers are present in this same sample. Therefore, western blots were used as the main method of monitoring enrichment since quantitative methods were not needed.

In our approach for subcellular fractionation, we used markers for endoplasmic reticulum (PDI), mitochondria (TOM20 for outer membrane and Tim23 for inner mitochondrial membrane), MAMs (FACL4), and plasma membrane (Na^+^/K^+^ ATPase). MAMs are comprised of both the mitochondria and endoplasmic reticulum. Therefore, the presence of a small quantity of MAMs in the ER fraction is to be expected. However, a large quantity of MAMs in the ER section would indicate a contaminated, non-pure reticulum fraction.

## Validation of protocol

**Figure 8.**
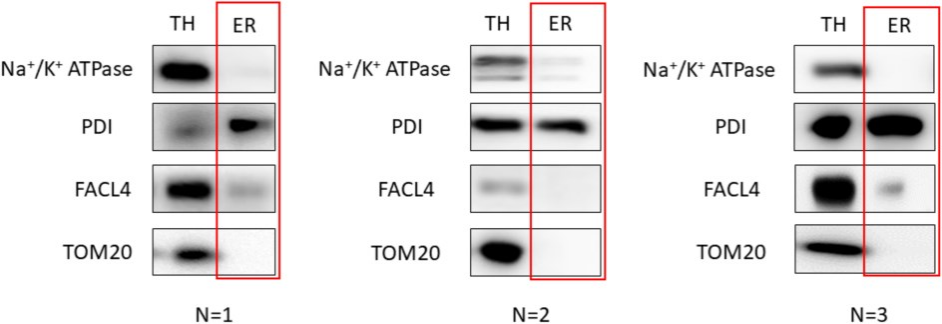
Validation of the protocol using HeLa cells

**Figure 9.**
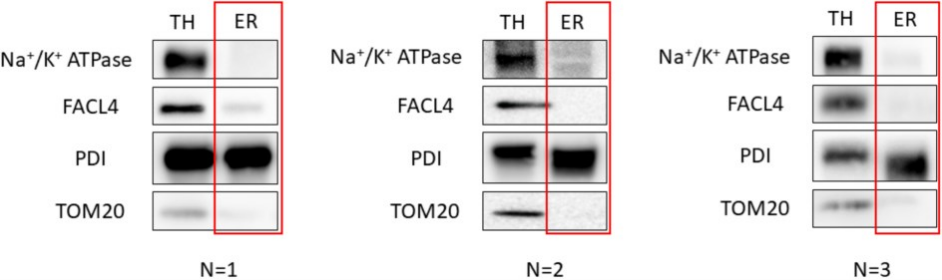
Validation of the protocol using Mouse Liver

To achieve reproducibility, we fine-tuned the protocol by producing three replicates of our results. Additionally, three independent isolations on different days were performed to ensure the robustness of the protocol. The western blot analysis images presented previously in this protocol correspond to the optimal results achieved. These can be observed below for HeLa cells (Figure 8) and mouse liver (Figure 9) extracts:

## General notes and troubleshooting

**General notes**

1. In the case of the subcellular fractionation performed with cells, the quantity of reticulum obtained is approximately 300 μL for approximately 2.5 μg/μL. This amount can be sufficient for some analysis, such as checking the presence of some other proteins in that organelle, but might not be enough for more complex analysis.
2. All the buffers must contain a protease inhibitor cocktail and each sample must be kept at 4 °C (or on ice) to avoid degradation. For this reason, every centrifugation must be done at 4 °C as well.
3. The liver protocol was performed in mice fed with a normal diet. The results of this protocol can vary when mice are treated with different diets, especially those that cause alterations in the lipidic composition of the liver. Usually, 500 μL at 15 μg/μL of ER is obtained.
4. This protocol uses a fixed-angle rotor. We recommend the use of these types of rotors for these purposes, even though other models can also be used. Note that different rotors may alter the quality of the purification.

**Troubleshooting**

1. Cell confluence is very important and must be approximately 80%–90%; otherwise, the amount of sample obtained can be severely compromised. To solve this issue, one more dish can be added in case the others are not so confluent, in order to obtain an amount similar to what would be obtained with five dishes.
2. The results may change depending on the state of the cells. Cells with a high passage or that are undergoing senescence can cause different outcomes from the ones shown in this protocol. The recommended passage of HeLa cells should be approximately 1–80.
3. If an abnormal contamination in the fractions is observed, the problem may rely on the homogenization step. The speed, strength, temperature, and uniformity of homogenization and bubble formation during this process are common causes for this issue. Also, note that these characteristics as well as the number of strokes can be adapted for any specific laboratory conditions.
4. If, after the two low speed centrifugations (step 4 for the HeLa cells part, or 2a and 2b for the mouse liver part), a larger pellet is observed, the supernatant can be centrifuged again to clean the sample even more.
5. In the case of abnormal plasma membrane contamination in HeLa cell fractionation, an additional centrifugation can be performed just after step A5a following these same parameters.
6. Abnormal contamination can also occur if the supernatant is taken using a very thin tip. To avoid unwanted breakage, it is ideal to handle the supernatants with a tip that has a diameter of approximately 0.5 mm. Pellets that will be re-used for further purification steps should also be handled with these types of tips.
7. In most cases, the degree of contamination that the final result shows is mainly due to a bad homogenization. Ensure no bubbles are formed during this step and everything is performed on ice to avoid degradation. To improve this step, the number of strokes can be fine-tuned by homogenizing the cells and checking the degree of breakage using trypan blue stain and observing it under the microscope. A good degree of breakage can be considered when approximately 90% of the cells observed are broken.
8. After the homogenization, when the cells or the tissue homogenate are centrifuged, if a big pellet is observed, this can mean that there is a big volume of cells that has not been properly homogenized. In this case, the pellet can be resuspended using the same buffer as it was before the homogenization and a second homogenization can be done. The result of this second homogenization can be either added to the first one or you can perform the protocol in parallel with the first one and join the final pellets. This second way has been observed to be more successful, even though no exhaustive analysis has been done to prove its efficacy with certainty.
9. Some of the pellets can be a bit looser; for this reason, a lack of a band in the western blot analysis can be due to an accidental aspiration of some of the pellet.
10. If low yield is observed, this may be due to a low amount or bad quality of starting material (stressed cells, tissue undergoing degradation, apoptotic cells, …).
11. As a troubleshooting technique, the cell debris can be analyzed under the microscope or via western blot to determine if the homogenization was performed correctly.
